# Systems-Level Feedbacks of NRF2 Controlling Autophagy upon Oxidative Stress Response

**DOI:** 10.3390/antiox7030039

**Published:** 2018-03-05

**Authors:** Orsolya Kapuy, Diána Papp, Tibor Vellai, Gábor Bánhegyi, Tamás Korcsmáros

**Affiliations:** 1 Department of Medical Chemistry, Molecular Biology and Pathobiochemistry, Semmelweis University, Tűzoltó utca 37–47, 1094 Budapest, Hungary; banhegyi@eok.sote.hu; 2 Department of Genetics, Eötvös Loránd University, Pázmány Péter sétány 1/C, 1117 Budapest, Hungary; pappdia@gmail.com (D.P.); vellai.tibor@gmail.com (T.V.); Tamas.Korcsmaros@earlham.ac.uk (T.K.); 3 Genetics Research Group, Hungarian Academy of Sciences, Eötvös Loránd University (MTA-ELTE), Pázmány Péter stny. 1/C, 1117 Budapest, Hungary; 4 Pathobiochemistry Research Group, Hungarian Academy of Sciences, Semmelweis University (MTA-SE), Tűzoltó utca 37–47, 1094 Budapest, Hungary; 5 Gut Health and Food Safety Programme, Quadram Institute, Norwich Research Park, Norwich NR4 7UA, UK; 6 Earlham Institute, Norwich Research Park, Norwich NR4 7UZ, UK

**Keywords:** oxidative stress, autophagy, mathematical modeling, feedback loop, oscillation

## Abstract

Although the primary role of autophagy-dependent cellular self-eating is cytoprotective upon various stress events (such as starvation, oxidative stress, and high temperatures), sustained autophagy might lead to cell death. A transcription factor called NRF2 (nuclear factor erythroid-related factor 2) seems to be essential in maintaining cellular homeostasis in the presence of either reactive oxygen or nitrogen species generated by internal metabolism or external exposure. Accumulating experimental evidence reveals that oxidative stress also influences the balance of the 5′ AMP-activated protein kinase (AMPK)/rapamycin (mammalian kinase target of rapamycin or mTOR) signaling pathway, thereby inducing autophagy. Based on computational modeling here we propose that the regulatory triangle of AMPK, NRF2 and mTOR guaranties a precise oxidative stress response mechanism comprising of autophagy. We suggest that under conditions of oxidative stress, AMPK is crucial for autophagy induction via mTOR down-regulation, while NRF2 fine-tunes the process of autophagy according to the level of oxidative stress. We claim that the cellular oxidative stress response mechanism achieves an incoherently amplified negative feedback loop involving NRF2, mTOR and AMPK. The mTOR-NRF2 double negative feedback generates bistability, supporting the proper separation of two alternative steady states, called autophagy-dependent survival (at low stress) and cell death (at high stress). In addition, an AMPK-mTOR-NRF2 negative feedback loop suggests an oscillatory characteristic of autophagy upon prolonged intermediate levels of oxidative stress, resulting in new rounds of autophagy stimulation until the stress events cannot be dissolved. Our results indicate that AMPK-, NRF2- and mTOR-controlled autophagy induction provides a dynamic adaptation to altering environmental conditions, assuming their new frontier in biomedicine.

## 1. Introduction

The key role of multicellular organisms is to maintain their inner protein homeostasis, also called proteostasis, against various harmful environmental factors (such as nutrient deprivation and oxidative attacks). From internal metabolism and external toxicant exposure, reactive oxidants might be formed generating oxidative stress in the cell. Reactive oxidants include reactive oxygen and nitrogen species (ROS and RNS, respectively), such as O_2_^–^, H_2_O_2_, NO, NO_2_ [1]. These highly reactive molecules are formed in a controlled manner even at physiological conditions; however hyper-production of oxidative agents might have fatal consequences by inducing cancer development, chronic diseases and toxicity [2]. Therefore cellular oxidative stress response mechanisms have to be highly controlled [3]. It is well known that mimicking oxidative stress by H_2_O_2_ or *t*BHP (*terc*-butyl-hydroperoxide) treatment results in activation of several signal transduction pathways of that stress response mechanism; meanwhile the ongoing cell division cycle must be blocked [4].

The nuclear factor erythroid 2-related factor 2 (NRF2) has a key role to enable cell adaptation to oxidative stress [5,6]. NRF2 is a member of the cap ‘n’ collar (CNC) subfamily of basic region leucine zipper (bZip) transcription factors [7]. It is rapidly degraded by proteasomes at physiological conditions with a half-life of 20 mins [5,6]. In this process, NRF2 is regulated in the cytoplasm by CUL3/RBX1-dependent E3 ubiquitin ligase (KEAP1 complex) [8]. This complex has an essential role in facilitating the ubiquitination and subsequent proteolysis of NRF2 [8]. Various oxidative agents can modify cysteine residues on KEAP1, resulting in its conformational changes and thereby preventing the degradation of newly synthesized NRF2. This process results in a quick accumulation of NRF2 level in the cell upon oxidative stress [9]. Therefore NRF2 is able to translocate to the nucleus where it becomes transcriptionally active [10]. 

The induction of NRF2 genes requires a common NRF2-binding motif on the DNA, known as antioxidant response element (ARE) or electrophile response element (EpRE) [11]. The active NRF2 transcriptionally controls more than 2000, mainly cytoprotective genes [12]. NRF2 also directly reduces the levels of both ROS and RNS by promoting the expression of their suppressors (i.e., catalase or nitric oxide synthase) [6]. Furthermore, NRF2 induces the expression of other protective genes encoding phase I and II detoxification enzymes, transport proteins, proteasome subunits, the regulators of various cell death mechanisms, antioxidant proteins, growth factors, chaperones and other transcription factors [13,14]. 

It has been already shown that the cellular oxidative stress response mechanism also involves the activation of (macro)autophagy [15]. “Autophagy” in Greek is also called “self-eating” referring to its ability to “digest” injured parts of the cytoplasm and intracellular organelles, assuming its cytoprotective role. During macroautophagy (hereafter referred as to autophagy), cellular components get sequestered into a vesicle termed autophagosome, contents of which are delivered for degradation to the lysosomal compartment (the fusion of an autophagosome with a lysosome forms an autolysosome) [16,17]. Cells have some basal autophagic activity under physiological conditions; however this self-digesting process gets more efficient upon various stress events [18]. Interestingly, excessive levels of autophagy might induce cell death in apoptotic-deficient cells [16,17].

The induction of autophagy requires a complex signal transduction pathway, containing many genes (most of them are named autophagy related genes or *ATG*s for short) [19]. Autophagy is strictly controlled by the two sensors of cellular homeostasis, mTOR and AMPK [20,21,22]. The mTOR (mammalian target of rapamycin) pathway is a master regulator of cellular proteostasis by integrating inputs from external and internal signals, such as growth factors, amino acids, glucose and energy status, and therefore controls growth and metabolism including translation and ribosome biogenesis [23]. AMPK (AMP activated protein kinase) has a crucial role in maintaining energy homeostasis by sensing cellular energy status [24]. AMPK tightly controls ATP-consuming processes, such as glycogen or protein synthesis, and up-regulates processes that yield ATP (i.e., glycolysis) [25,26]. The mTOR- and AMPK-dependent autophagy regulation is achieved through ULK-1 (Unc-51-like autophagy activating kinase), a Ser/Thr protein kinase, which is a key molecule of an autophagy inducer complex [27]. Besides ULK-1, the complex contains several other proteins, such as Atg13, Atg101 and FIP200 [28]. 

NRF2 regulation is linked to autophagy modulation via p62, an autophagy substrate and cargo receptor. p62 (also known as SQSMT1; sequestosome) targets proteins to be transferred to autophagosome, and therefore promotes their autophagy-dependent degradation [29]. Oxidative stress induces p62 phosphorylation, which distinctly increases its binding affinity to KEAP1 [30]. p62-binding to KEAP1 quickly destroys the NRF2-KEAP1 complex, resulting in the dissociation of active NRF2 from KEAP1. Since KEAP-p62 complex rapidly gets degraded by autophagy, NRF2 is suggested to be positively regulated upon oxidative stress [31,32]. Moreover, NRF2 activates the expression of many autophagy genes, such as *Atg3*, *Atg5*, *Atg7* and *SQSTM1* during cellular stress response [33,34]. It has been also shown that NRF2 deficiency impairs autophagy, therefore NRF2 seems to have an essential positive effect on the autophagic process (unpublished data).

In the current study, we aimed to explore the comprehensive role of NRF2 upon oxidative stress. To address this issue, we viewed the available experimental data form theoretical perspective by creating a mathematical model. We found that NRF2, AMPK and mTOR form an incoherently amplified negative feedback loop, claiming that NRF2 is the key molecule to fine-tune autophagy according to the level of oxidative stress. We also show that different levels of oxidative stress sensed by NRF2 can generate various cellular responses, periodically repeating autophagy, or cell death.

## 2. Materials and Methods

### Introducing the Mathematical Model of NRF2-Induced Oxidative Stress Response Mechanism

In this study, a systems biological method is used to understand the dynamical features of the NRF2-induced oxidative stress response mechanism. A system-level view can be developed by bringing together the main components and their interactions reported in the literature. A biological network can be translated into a set of ordinary differential equation (ODE) based on biochemical reaction kinetics. An ODE describes how each component level/activity in the network changes with the time. A generic differential equation depicting the temporal changes of a regulatory component (called protein X_a_) is composed of two parts: production and consumption terms. The production can be given by protein synthesis and/or an activation term, while the consumption can be given by protein degradation and/or inactivation term [35].

dX_a_/dt = k_s_ + k_act_ × (X_T_ − X_a_) − (k_d_ + k_in_) × X_a_ (1)

where

X_a_—concentration of active X;
X_T_—total concentration of X;
k_s_—synthesis rate constant of X;
k_act_—activation rate constant of X_a_;
k_d_—degradation rate constant of X;
k_in_—inactivation rate constant of X_a_.

Usually synthesis and degradation reactions are described by mass action kinetics, whereas protein activity can be described either by mass action or Michaelis-Menten kinetics [36,37]. The rate constants (*k*) have the dimension of min^−1^ and Michaelis constants (*J*) are dimensionless. The proteins levels/activities are given in arbitrary units (a.u.). The exact value of the various parameters (i.e., rate constants, Michaelis constants) and initial conditions must be specified to solve ODEs. Since the biological system has non-linear nature, it makes difficult to find the solution of ODEs analytically. To describe the dynamical features of the control network precisely, the equations must be solved numerically. The dynamical features of the control network can be characterized by either time courses or phase plane analysis or signal response curves [35,36,38].

The time evolution of the protein activity and/or levels of the key components will be studied by implementing time courses. The time courses have been calculated by numerical integration of the full set of our differential equation system of contained by our model. 

To understand characteristic dynamical features of the regulatory network and its dependence on its parameter values, two-dimensional phase plane analysis will be carried out. In two-dimensional phase plane analysis, the two slowest variables are followed, while fast variables are separated and assumed to be already in steady state. The kinetic behaviors of the differential equations can be visualized graphically by balance curves on phase plane portrait. The balance curve of a protein level or activity denotes where the rate of the protein production and consumption terms are balanced. This method represents the observable physiological states of the cell cycle regulatory system. The intersections between two balance curves are called equilibrium points: here the system has steady-state solutions.

Plotting signal response curves are used to understand the dynamic changes in the nonlinear system as a function of a specific parameter. A single variable is chosen from the complex regulatory system which is supposed to characterize all interacting proteins in the network. Moreover, the selected parameter may represent the main effect involved in these relations. The behavior of the system can be graphically analyzed on signal response curves. The stable solutions with solid lines, the unstable solutions with dashed lines are depicted, while the dots assume oscillatory characteristic on signal response curves.

Dynamical simulations were carried out using the program XPPAUT (Department of Mathematics, University of Pittsburgh, Pittsburgh, Pennsylvania, USA), which is freely available from http://www.math.pitt.edu/~bard/xpp/xpp.html. We provide the XPP codes that can be used to generate all the figures in the manuscript (see the Supplementary Material Document S1).

## 3. Results

### 3.1. The Regulatory Network of NRF2 is Highly Similar to ULK-1-Controlled Cellular Stress Response Mechanism

**Figure 1:**
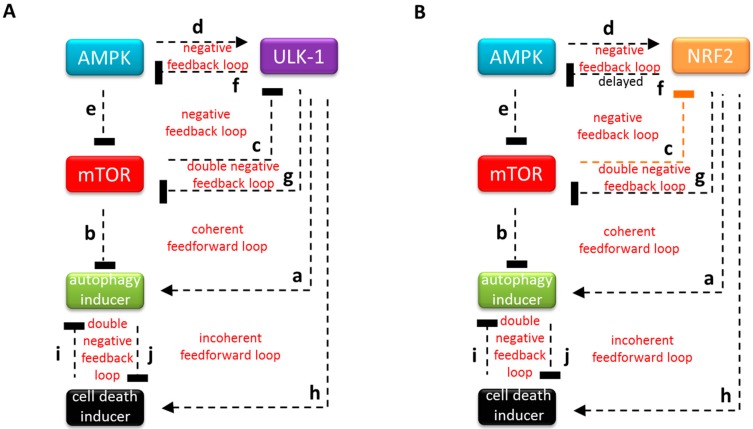
The key regulatory components and their interactions at various stress events. The wiring diagram of (**A**) (Unc-51-like autophagy activating kinase) ULK1-regulated stress response mechanism during starvation (**B**) and nuclear factor erythroid-related factor 2- or NRF2-controlled oxidative stress response mechanism. Autophagy inducer refers to those elements which can induce autophagy with respect to cellular stress, while cell death inducer means the sum of the activators of cellular cell death process. Dashed lines show how the molecules can influence each other. Blocked end lines denote inhibition. The experimentally unproven connection is orange-colored. AMPK: 5′ AMP-activated protein kinase; mTOR: mammalian kinase target of rapamycin.

Taking into consideration recent experimental results, we could build up the controlling network of starvation-induced response mechanism (Figure 1A). Namely, it has revealed that ULK-1 protein has a comprehensive role during nutrient depletion. ULK-1 is a serine/threonine kinase, and one of the key subunits of the autophagy inducer complex [27] (Figure 1A a). It is already well known that the proper balance of mTOR and AMPK kinases is crucial for determining whether autophagy is active or not upon cellular stress. mTOR is the central member of a complex called mTORC1, and has a key role in guaranteeing appropriate protein synthesis under physiological conditions, meanwhile the autophagic process is completely blocked [23] (Figure 1A b). In contrast, AMPK maintains the cellular AMP/ATP ratio by re-balancing the cellular energy level via activation of autophagy upon stress events [39]. ULK-1 is regulated by an mTOR-dependent inhibitory phosphorylation under nutrient rich conditions [24] (Figure 1A c), however AMPK promotes the autophagic process by phosphorylating ULK-1 upon cellular stress [21,24] (Figure 1A d). To make sure that autophagy gets fully activated during cellular stress, AMPK activates ULK-1 and inhibits mTOR both directly and indirectly. Namely, AMPK blocks mTOR by phosphorylating Tuberous Sclerosis Complex 2 (TSC2) [40], an inhibitor of mTOR complex, and also inhibits one subunit of the complex, Raptor [41] (Figure 1A e). In addition, ULK-1 can down-regulate both mTOR and AMPK via phosphorylation generating a sophisticated regulatory loop in the control network [24] (Figure 1A f and g). Two negative feedback loops (i.e., AMPK -> ULK-1 ┤ AMPK and ULK-1 ┤ mTOR ┤ AMPK ┤ ULK-1) are formed (Figure 1A). Nazio and colleagues have recently revealed that fine-tuning of both mRNA and protein levels of ULK-1 is essential for autophagy oscillation during starvation [42]. Interestingly, it was also shown that, beside ULK-1-dependent autophagy induction, sustained ULK-1 can promote cell death. Moreover, Joshi and colleagues have demonstrated that during excessive levels of oxidative stress, ULK-1 is able to induce a suicide cascade through PARP-1 [43] (Figure 1A h), suggesting that ULK-1 kinase has an important role in cellular life-and-death decision. 

However, it is well known that NRF2 senses oxidative agents and regulates antioxidant defense by promoting the transcription of a large amount of mainly cytoprotective genes. Therefore, we first explore the control network of NRF2 in oxidative stress response mechanism. Based on molecular biology data presented by us and other researchers, we can suggest that the crucial element of oxidative stress response mechanism controlled by NRF2 is relatively similar to that of ULK-1 during nutrient deprivation (Figure 1B).

It has been recently shown that AMPK promotes the nuclear accumulation of NRF2 by directly phosphorylating its Ser550 residue [44] (Figure 1B d). During cellular stress, NRF2 positively regulates the expression of many autophagy genes, such as *Atg3*, *Atg5*, *Atg7*, *SQSTM1* and *GABARAPL1* [34] (Figure 1B a), suggesting its positive role in autophagy induction. However it has been also shown that permanent oxidative stress results in a suppression of AMPK level suggesting that autophagy gets inhibited [45]. The NRF2ome, an online resource developed by us, provides an integrated systems-level database for NRF2 [46]. NRF2ome contains both experimentally verified and predicted interactions of NRF2. This database lists NRF2 as a regulator of AMPK. An NRF2-dependent control of AMPK was also suggested in *C. elegans* [47] .In addition we have recently directly presented that NRF2 has a negative effect on AMPK in both human cell lines and *C. elegans* (unpublished data) (Figure 1B f). Therefore, we claim that NRF2 also has an indirect negative effect on autophagy via down-regulating AMPK expression. In our model we suppose that NRF2 as a transcriptional repressor binds to and inhibits AMPK mRNA synthesis. Therefore, NRF2 has a delayed negative effect on AMPK generating a negative feedback loop between NRF2 and AMPK upon sustained oxidative stress (unpublished data). Besides inhibiting AMPK, NRF2 also controls mTOR activity. Although some data have suggested that NRF2 promotes mTOR pathway in cancer cells [48], we did not detect any mTOR activity when NRF2 was present during oxidative stress (unpublished data). This observation is a logical consequence of that fact that autophagy must be active during oxidative stress; its inhibitory effect controlled by mTOR must therefore be diminished. We claim that NRF2 has a negative effect on mTOR pathway (Figure 1B g). Bendavit and colleagues have shown that NRF2 binds to the ARE sequence of mTOR promoter region, thereby confirming a direct NRF2-dependent regulation on mTOR pathway [49]. As already suggested in *C. elegans*, NRF2 is negatively regulated by mTOR [50] (Figure 1B c), which generates a double negative feedback loop between NRF2 and mTOR in our control network. An extra negative feedback loop should be formed which contains a negative regulatory triangle of NRF2, mTOR and AMPK (Figure 1B). Although NRF2 has a crucial role in inducing autophagy-dependent cell survival, it has recently shown that NRF2 also enhances cell death upon oxidative stress [51] (Figure 1B h). This prompted us to assume that NRF2 also promotes cell death mechanisms. We suppose that a mutual antagonism should operate between autophagy and cell death inducers, generating a double negative feedback loop in the control network [52] (Figure 1B i and j). Based on these data, we can suggest that NRF2 regulation is homologous to the control network of starvation-induced ULK-1, supposing its important regulatory role in oxidative stress response mechanism.

### 3.2. Systems-Level Feedbacks Guarantee a Proper Answer to both Tolerated and Unbearable Oxidative Stress Factors

**Figure 2:**
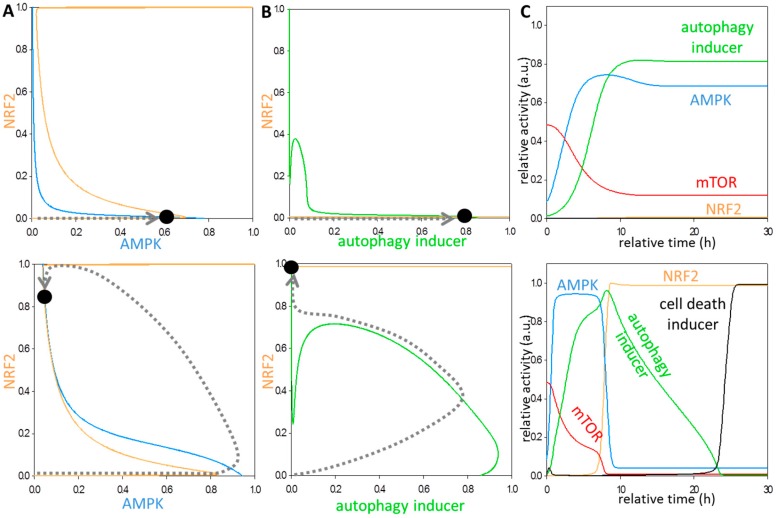
The characteristic properties of control network at various levels of oxidative stressor. (**A**) AMPK-NRF2 and (**B**) autophagy inducer-NRF2 phase plane diagrams at low (upper panel) and high (lower panel) levels of oxidative stress. The balance curves of AMPK (light blue), autophagy inducer (green) and NRF2 (yellow) are plotted. The phase planes are shown for (upper panel) stress = 0.1 and (lower panel) stress = 5. Intersections of nullclines represent the stable (filled circle) steady states. Trajectories are depicted with dotted grey lines. (**C**) The computational simulations are determined at low (upper panel) and high (lower panel) levels of oxidative stress. The dynamic simulation of the model with (upper panel) continuous low stress conditions (stress = 0.1) and (lower panel) continuous high stress conditions (stress = 5). The relative activity of mTOR, AMPK, NRF2 and autophagy inducer is shown.

To illustrate the qualitative features of oxidative stress response mechanism, we present a simple mathematical model based on Figure 1B (for detailed description of the mathematical model see the Supplementary Material Document S1). The reaction kinetic equations are written for the rate change of each component, generating a non-linear differential equation system. The dynamical characteristic of these non-linear differential equations can be appropriately illustrated in a coordinated system spanned by AMPK and NRF2 (Figure 2A) or NRF2 and autophagy inducer (Figure 2B). Autophagy inducer refers to the active form of a complex, which is essential to turn on autophagy upon cellular stress. If the activity of autophagy inducer is high, the cell is supposed to be under an intensive autophagic process. If all the other elements are in steady state, we can plot the balance curves for NRF2, AMPK and autophagy inducer (see the yellow, blue, and green curves on Figure 2A,B, respectively). Along the balance curve, the rate of synthesis/activation is exactly balanced by its rate of degradation/inactivation of the given component. The intersections between two balance curves are called equilibrium points: here the system has steady state solutions, representing the observable physiological states of the regulatory system. Under physiological conditions, in both cases the balance curves intersect at close to zero, referring that NRF2, AMPK are down-regulated, while autophagy has some basal activity when conditions are optimal for the cell (figures not shown).

Depending on the level of oxidative stress, cells can induce autophagy either permanently or temporarily. At low levels of oxidative stress, the intersection of stable states moves to the right on the phase plane diagrams (see Figure 2A,B, upper panel). The left of the blue/green curve AMPK/autophagy inducer is increasing, while to the right it is decreasing on both curves. Similarly, below the yellow curve NRF2 is increasing and above the curve it is dropping. The “folded” character of NRF2 and autophagy inducer balance curves is the consequence of the positive (i.e., double negative) and negative feedback loops of the control network. At low levels of oxidative stress, the intersection refers to a stable steady state with negligible amounts of NRF2 (Figure 2A,B, upper panel) and intermediate levels of both autophagy inducer (Figure 2B, upper panel) and AMPK (Figure 2A, upper panel). In case of excessive levels of oxidative stress, either AMPK or autophagy inducer balance curves move upwards (Figure 2A,B, lower panel), resulting in a displacement of the stable state in the control network. At high levels of stress, NRF2 seems to be fully active meanwhile both autophagy inducer and AMPK get down-regulated. To explain the oxidative stress response mechanism at excessive levels of stress, we plotted the trajectories on the control planes (Figure 2A,B, lower panel). Interestingly, both autophagy and AMPK show a transient activation (via remembering to the autophagic steady state), but later the system finds a stable state with relatively low levels of AMPK and autophagy inducer. These results suggest that autophagy will be temporarily active even at high levels of oxidative stress, but later it gets inhibited. 

To further confirm the dynamical characteristic of the control network, we present numerical solutions of the kinetic equations at both low and high levels of oxidative stress (Figure 2C). Under physiological conditions, only mTOR is active which has an essential role in protein homeostasis. At modest levels of oxidative stress, mTOR activity drops since the increasing amount of AMPK can downregulate it (Figure 2C, upper panel). In the absence of mTOR, AMPK causes the induction of autophagy. Although AMPK has a positive effect on NRF2, its activity is not strong enough to turn it on, therefore NRF2 remains inactive. This result suggests that NRF2 is not essential for upregulating autophagy in this case. However, when the permanent oxidative stress reaches a threshold, the control system generates a completely different answer. Although AMPK induces autophagy and inhibits mTOR, now the autophagic process has only a transient activation peak (Figure 2C, lower panel). In this case, NRF2 has a delayed activation due to AMPK-dependent upregulation. The increasing amount of NRF2 however quickly inhibits its activator (i.e., AMPK), and therefore the activity of autophagy inducer is also dropping. Since NRF2 has a negative effect on mTOR, mTOR remains inactive even in the absence of AMPK. Parallel to the inactivation of autophagy, the cell death process becomes activated via a NRF2-dependent induction. The observed kinetic features of oxidative stress response mechanism are nicely related to our unpublished data.

These results suggest that the activation profile of NRF2 highly controls the dynamical characteristic of cellular life-and-death decision upon various levels of oxidative stress.

### 3.3. Each Element of the Regulatory Triangle Effectively Affects Autophagy Induction during Oxidative Stress

**Figure 3:**
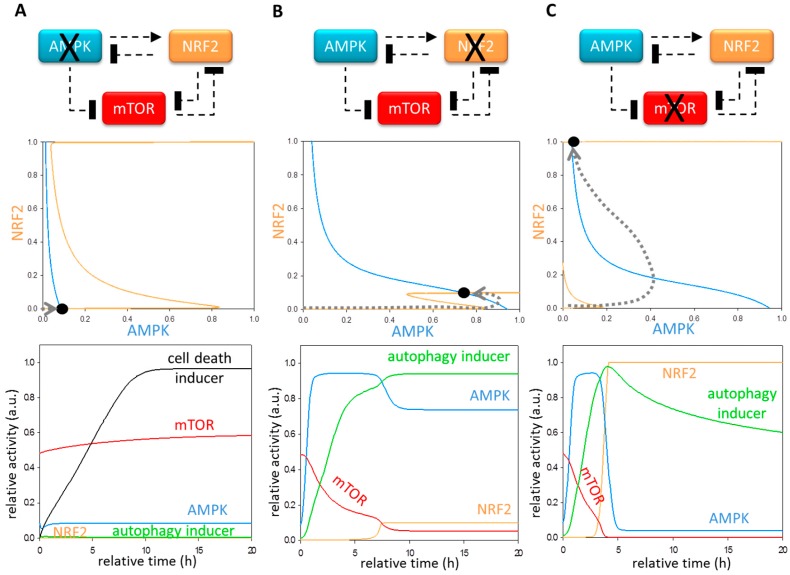
The crucial role of AMPK-NRF2-mTOR regulatory triangle in oxidative stress response mechanism. The effect of (**A**) AMPK (**B**) NRF2 and (**C**) mTOR deprivation was systematically analyzed upon simulating high levels of oxidative stress (stress = 5). Dashed line shows how the molecules can influence each other. Blocked end lines denote inhibition. Phase plane diagrams are plotted upon excessive levels of oxidative stress (upper panel). The balance curves of AMPK (light blue) and NRF2 (yellow) are plotted. The phase planes are shown for (**A**) AMPKT = 0.1, (**B**) NRF2T = 0.1 or (**C**) mTORT = 0.1 when stress = 5. Intersections of nullclines represent the stable (filled circle) steady states. Trajectories are depicted with dotted grey lines. The computational simulations are determined upon excessive levels of oxidative stress (lower panel). The temporal dynamics is simulated with high stress (stress = 5) combined with (**A**) AMPK down-regulation (AMPKT = 0.1), (**B**) NRF2 down-regulation (NRF2T = 0.1) or (**C**) mTOR down-regulation (mTORT = 0.1) when stress = 5. The relative activity of mTOR, AMPK, NRF2, autophagy and cell death inducers is shown.

Next, we investigated the exact role of each element of the regulatory triangle (i.e., AMPK, NRF2 and mTOR) in autophagy-induced oxidative stress response mechanism. The regulation of AMPK seems to be contradictory. It is well known that AMPK is activated upon oxidative stress to enhance autophagy; however, our recent experimental data revealed that NRF2 has a delayed negative effect on AMPK. To explore the importance of these opposite control effects on AMPK, both AMPK upregulation and downregulation is further followed by theoretical techniques. When AMPK is overexpressed upon excessive levels of oxidative stress, NRF2 gets quickly activated, but is not able to downregulate the hyper-activated AMPK, leaving autophagy active (figures not shown). To further confirm the role of AMPK in autophagy induction, excessive levels of oxidative stress were induced in cells in the absence of active AMPK. In this case, the system has a steady state when both NRF2 and autophagy inducer are inactivated, suggesting that AMPK has a key role in NRF2 activation (Figure 3A). Depletion of AMPK prevents mTOR downregulation even at high levels of oxidative stress (Figure 3A, lower panel). Since autophagy remains inactive, the cell death mechanism can quickly switch on. These results assume that AMPK is crucial to promote autophagy-dependent cell survival; meanwhile it also turns on its own delayed suicide mechanism via NRF2 induction during excessive levels of oxidative stress.

Next, we examined the effect of NRF2 depletion upon high levels of oxidative stress (Figure 3B). The absence of NRF2 does not affect the activation profile of AMPK, generating a proper autophagy induction, while mTOR gets downregulated. However, NRF2 is not present in the cell, thus AMPK becomes quickly hyper-activated, leading to an abnormally intensive autophagic process. Simulation data match to our experimental results, when NRF2 silencing in human cells caused a permanent autophagic response upon excessive levels of oxidative stress (unpublished data). Since constantly elevated levels of autophagy might be fatal for the cell, we can conclude that NRF2 has a key role in terminating autophagy via AMPK downregulation. These results also suggest that while AMPK is essential to switch on autophagy, NRF2 works as a most important guardian against the aberrantly high autophagy-induced cell death by inhibiting AMPK.

Since mTOR inhibits both autophagy inducer and NRF2, downregulation of mTOR pushes NRF2 balance curves to left on the phase plane, suggesting that NRF2 gets activated much easier during oxidative stress (Figure 3C, upper panel). Computational simulation shows that AMPK induction followed by autophagy activation is exactly the same in the absence and presence of mTOR (Figure 3C, lower panel). However, NRF2 quickly gets induced, and inhibits AMPK. Furthermore, autophagy remains active in the absence of mTOR. Corresponding to our computational data, Wang et al., have recently proposed that both NRF2 and autophagy become hyper-activated when mTOR is downregulated by rapamycin treatment [53]. Our results suppose that mTOR has an essential role to help NRF2 to keep autophagy in an inactive state at the later phase of excessive levels of oxidative stress. 

### 3.4. The NRF2-AMPK-mTOR Regulatory Loop Generates a Sustained Oscillation upon Intermediate Stress Levels

It is well known that a negative feedback loop can generate a sustained oscillatory response if the feedback loop has a certain delay, i.e., the regulatory network contains at least three components. Since in the heart of the control network an AMPK ┤ mTOR ┤ NRF2 ┤ AMPK negative feedback loop exists, a question immediately arises of whether an oscillatory response can be observed or not upon oxidative stress. Furthermore, we have recently shown that the NRF2-induced negative effect achieves a delayed downregulation of AMPK expression. This time lag generated by NRF2-dependent regulation is based on the fact that NRF2 as a transcription factor binds to the AMPK mRNA, and presumably hinders AMPK protein synthesis. Therefore, in our model the time-delay is achieved by an indirect NRF2-dependent inhibition of AMPK protein. This might further help guarantee a periodically repeating cellular response. A double negative feedback can be also detected between mTOR and NRF2, generating a so-called incoherently amplified negative feedback loop (Figure 1B).

**Figure 4:**
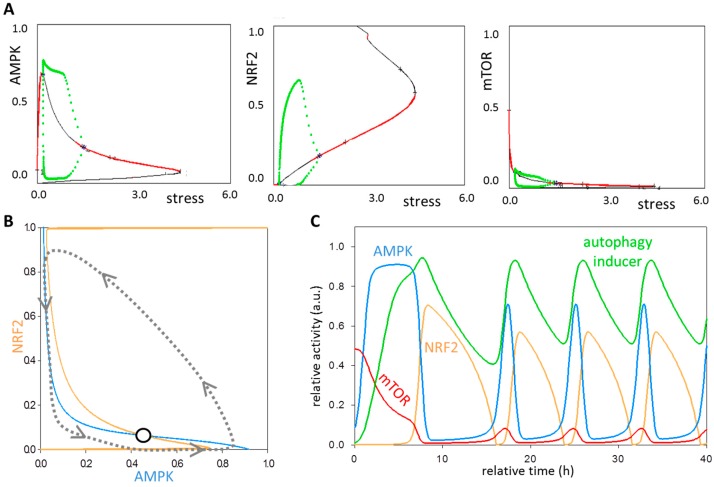
The oscillatory characteristic of the oxidative stress response mechanism. (**A**) The bifurcation diagrams of AMPK (panel left), mTOR (panel middle) and NRF2 (panel right). The signal response curves are shown with respect to the increasing stress level. Solid red lines denote stable states, while solid black line denotes the unstable state. Green dots around the unstable states represent oscillatory behavior. (**B**) Phase plane diagram at intermediate levels of oxidative stress. The balance curves of AMPK (light blue) and NRF2 (yellow) are plotted. The phase plane is shown for stress = 0.5. Intersection of nullclines represent the unstable (open circle) steady state. The trajectory is depicted with dotted grey line. (**C**) The computational simulations are determined at intermediate levels of oxidative stress (stress = 0.5). The relative activity of mTOR, AMPK, NRF2 and autophagy inducer are shown.

To reveal the oscillatory characteristic of the control network upon oxidative stress, the stress level was systematically increased, meanwhile the presence of either active AMPK, NRF2 or mTOR was detected (Figure 4A). Each AMPK, NRF2 and mTOR bifurcation diagram contains a range (between stress = 0.2 and 1.5) with well-defined boundaries when an oscillatory behavior is performed. Therefore, when we assume intermediate signal strengths, the system executes oscillation.

To further prove that the regulatory system has a sustained oscillation, the balance curves of AMPK and autophagy inducer were plotted at a chosen intermediate stress value (Figure 4B). The two curves now intersect on one sate, but this is unstable state and a stable oscillation can be observed around it. The delayed, three-component negative feedback loop alone might be able to perform oscillatory characteristic, however the additional double negative feedback between NRF2 and mTOR results in folded balance curves adding bistability to the control mechanism. Although both low and high levels of oxidative stress result in stable steady states (i.e., intensive autophagy or induction of cell death), the feedback loop causes that the control system repeatedly overshoot or undershoot its steady state at intermediate stress levels (see the dashed trajectory on Figure 4B).

Computational simulations showed a sustained oscillation for the four-component loop at intermediate levels of oxidative stress (Figure 4C). In case of oxidative agents, AMPK gets quickly activated followed by autophagy inducer. However, the increasing amount of NRF2 can block AMPK gene expression resulting in a drop of AMPK protein level with a certain time delay. NRF2 cannot be stabilized itself at this level of oxidative stress, because mTOR quickly comes back and kills it. NRF2 downregulation causes a reactivation of both AMPK and autophagy inducer. Our results suggest that the regulatory system can oscillate, generating a periodically repeating autophagy induction upon intermediate levels of oxidative stress.

### 3.5. The Importance of NRF2-mTOR Double Negative Feedback Loop

In the regulatory network, NRF2, which acts as the inhibitor of AMPK, is amplified by a positive feedback loop. Namely, a mutual antagonism is observed between mTOR and NRF2. It has been already confirmed that mTOR is able downregulate NRF2, but the NRF2 ┤ mTOR regulatory interaction is doubtful. In this study, we assumed that mTOR can block NRF2 by inhibitory phosphorylation, based on fact that ULK-1 is also downregulated in the analogous way. We suggested the importance of mTOR-NRF2 double negative feedback loop in the control network by generating so called “kinks” in the balance curve (figure not shown). These “kinks” help achieve well-separated stable steady states at low and high levels of oxidative stress, and they also force the dynamical system to execute sustained oscillation at intermediate levels of oxidative stress.

**Figure 5:**
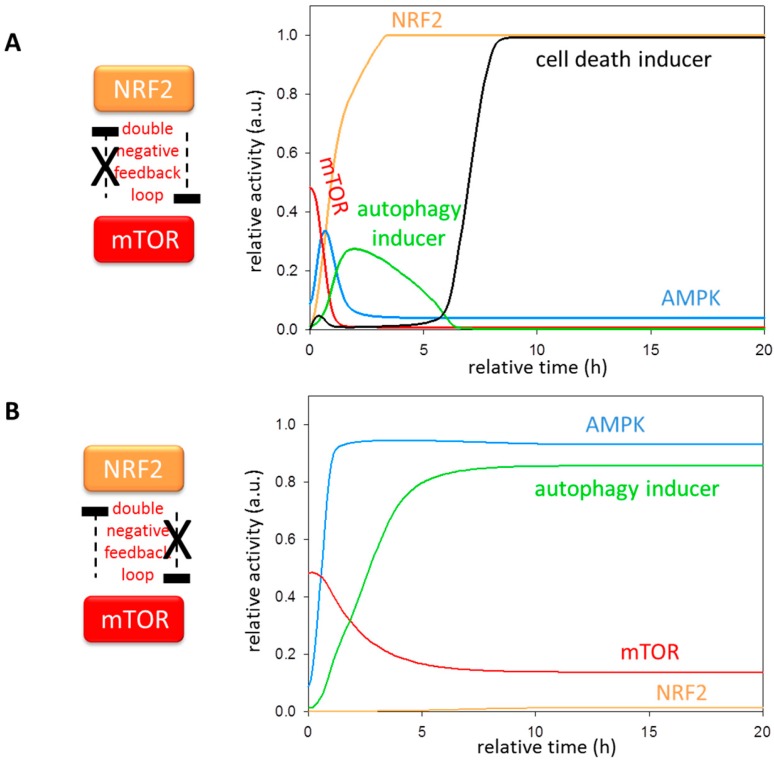
The crucial effect of mTOR-NRF2 double negative feedback loop in oxidative stress response mechanism. The computational simulations are determined in the absence of (**A**) mTOR ┤NRF2 or (**B**) NRF2 ┤ mTOR upon excessive levels of oxidative stress. The temporal dynamics is simulated with high stress (stress = 5) combined with (**A**) kinrf’ = 0 or (**B**) kimtor” = 0. The relative activity of mTOR, AMPK, NRF2, autophagy and cell death inducers is shown.

To further confirm the role of mTOR-NRF2 double negative feedback loop, we tested mutant phenotypes when the arms of the feedback loop was diminished systematically (Figure 5). If mTOR cannot inhibit NRF2, the latter can be fully active at much lower stress levels, leading to a premature inhibition of both AMPK and autophagy inducer. Since NRF2 also blocks mTOR, no mTOR can be detected. The cell death mechanism induced by NRF2 however turns on much earlier (Figure 5A). The absence of NRF2-dependent mTOR inhibition also has a drastic effect in the control network upon oxidative stress. Namely, mTOR activity remains high, therefore NRF2 cannot be active, generating hyper-active AMPK and autophagy inducer at excessive levels of stress (Figure 5B). These results suggest that AMPK alone is not sufficient for keeping mTOR in an inactive state, rather NRF2 is essential in this regulation. Together, these results imply that a double negative feedback loop between NRF2 and mTOR is crucial for adding bistability and robustness to the control mechanism.

## 4. Discussion

Various natural and artificial oxidative agents, such as ROS or H_2_O_2_, can drastically influence cellular homeostasis. While low levels of oxidative stress are fended off by inducing of autophagy-dependent survival, excessive levels of stress might cause cell death. It is well known that NRF2, as a transcription factor, can control the expression of many genes upon oxidative stress. However, its exact role in regulating the dynamical characteristic of life-and-death decision has not studied yet. To answer this question, we viewed the available experimental data form a theoretical perspective by creating a mathematical model. Taking into consideration the most important regulators and targets of NRF2 during oxidative stress, a comprehensive regulatory network was built (Figure 1B). We claim that the heart of the control network is an incoherently amplified negative feedback loop containing a delayed AMPK ┤ NRF2 ┤ mTOR ┤ AMPK negative feedback loop combined with a double negative feedback loop between mTOR and NRF2.

At physiological conditions mTOR is fully active, meanwhile autophagy-dependent self-cannibalism and AMPK are almost completely inhibited. According to the actual level of oxidative stress, this dynamical characteristic makes possible three different responses. In respond to modest oxidative stress, the control network generates a stable but mild autophagic response with active AMPK, meanwhile mTOR level drastically gets diminished and NRF2 is still inactive (Figure 2, upper panel). The systems-level feedbacks reveal that AMPK has a key role in inducing autophagy even at small-scale oxidative attack via mTOR downregulation. However, NRF2 remains inactive. In contrast, AMPK can activate NRF2 at excessive levels of oxidative stress, generating an even more intensive autophagic response, since NRF2 also activates the autophagy inducer (Figure 2, lower panel). An NRF2-induced negative feedback however can kill AMPK. Since NRF2 not directly down-regulates AMPK kinase, rather it represses *AMPK* expression, it creates a time delay in the regulatory loop. Although NRF2 has some activity to induce autophagy, NRF2 alone is not sufficient to maintain autophagic response. Therefore, autophagy-dependent survival has only a transient activity peak even at high levels of oxidative stress. Corresponding to our kinetic analysis (Figure 3), it has recently been shown that AMPK downregulation by its inhibitor (called Compound C) blocks autophagic response upon H_2_O_2_ treatment [54], while its chemical activator (called AICAR) suppresses the negative effects of oxidative stress [55]. The temporal profile of NRF2 predicted by our analysis must be investigated in the future.

Interestingly, between the two well-separated steady states of the control network (i.e., autophagic survival and cell death) there is an intermediate stress range when a sustained oscillation of AMPK, NRF2 and mTOR can be observed (Figure 4). This dynamical behavior generates a periodic autophagic response at intermediate oxidative stress (Figure 4). We argue that the most important role of autophagy is to help cell survival by degrading protein damage that is generated by oxidative stress. Although this self-eating is essential to protect cells from undergoing death, hyper-activated autophagy might also be harmful. We assume that uncontrolled autophagy would also digest proteins functioning properly. Therefore, the control network achieves a mild activity of autophagy inducer at low levels of oxidative stress, and also generates a pulsing autophagy at intermediate stress levels to make possible the monitoring of cellular conditions and switch off the process when circumstances get back to physiological conditions. Enduring oxidative stress quickly shuts off autophagy and induces an irreversible cell death mechanism. It is important to note that cell death never oscillates since its activation results in drastic irreversible modifications in the cell. Recently Nazzio and Cecconi have observed a periodically repeating ULK-1 and autophagy activation during starvation [42]. Their results fit our assumption that the dynamic regulation of NRF2- and ULK-induced autophagy is similar. While ULK-1 is essential during nutrient depletion, NRF2 seems to be crucial upon oxidative stress. Although this oscillatory behavior is already proved for ULK-1 [42], the periodic characteristic of the AMPK ┤ NRF2 ┤ mTOR ┤ AMPK incoherently amplified negative feedback loop needs further clarification.

Reviewing the wiring diagram shown on Figure 1B, we claim that only one connection of the regulatory triangle is predicted, namely mTOR can downregulate NRF2. Although this regulatory loop has shown in *C. elegans* [50], its presence is yet unknown in human cell lines. Here we showed that for the proper dynamical characteristic of the control network, a double negative feedback loop between NRF2 and mTOR seems to be essential. In the absence of the mTOR ┤ NRF2 regulatory arm, NRF2 has a premature activation upon oxidative stress (Figure 5). Consequently, NRF2-activation is independent from AMPK, resulting in its quick down-regulation before both AMPK and autophagy inducer could be activated. This double negative feedback loop is indispensable for its timing first to the system always having autophagy-dependent survival induced by AMPK, and only then to the switch on cell death with respect to excessive levels of oxidative stress. Therefore, we suppose that this double negative feedback loop stabilizes the two stable steady states via reinforcing bistability in the control network. Since mTOR is a protein kinase, we identified potential Ser phosphorylation sites on NRF2 with NetPhos 3.1 (Department of Bio and Health informatics, Technical University of Denmark, Lyngby, Denmark), a freely available software, to predict serine and threonine phosphorylation sites on the protein [56]. We found two potential Ser residues (Ser-351, Ser-356) of NRF2, and their phosphorylation motifs show high similarities to the phosphorylation site preferred by mTOR kinase (for details see the Supplementary Material Document S1). These analyses further suggest that mTOR might be able to phosphorylate these Ser residues on NRF2, supposing a regulatory connection between the two proteins. However, their presence and their directionality later must be proven experimentally.

It is intriguing that the orientation of NRF2-dependent regulation on its targets seems contradictory upon oxidative stress. NRF2 acts not only on a direct positive manner on autophagy induction, but it also has a negative effect on it via downregulating AMPK. NRF2 can enhance the inducers of both autophagy-dependent survival and cell death (Figure 1). Based on these regulatory loops, we assume that NRF2 is crucial for fine-tuning the cellular stress response mechanism. We propose that the proper timing driven by NRF2 is the key feature of the control network with respect to oxidative stress. At low levels of oxidative stress, AMPK promotes autophagy-dependent survival, while a transient activation peak of autophagy is also observable at excessive levels of oxidative agent. AMPK inactivation and the activation of cell death inducer by NRF2 guarantee that autophagy becomes inactive and cell death turns on, when the stress level reaches a critical value (Figure 2). At intermediate stress levels, NRF2 achieves an oscillatory characteristic of the control network, thereby generating periodically repeating activation of autophagy (Figure 5). NRF2 appears thus as the main switch molecule for cellular life-and-death decision upon oxidative stress.

It is well known that NRF2 transcription factor can promote the expression of several genes coding for autophagy inducers [33,34]. It also recently suggested that NRF2 promotes apoptosis [51]. We have previously collected various proteins controlling autophagy and apoptosis induction to study the regulatory mechanism at molecular level (see Figure 1 in [52,57]. These proteins might be potential targets of NRF2, too, hovewer these connections need further clarification. According to recently published experimental data we supposed a double negative feedback loop between autophagy and apoptosis inducers at various cellular stress events such as starvation and endoplasmic reticulum stress [52,57]. We also showed that this feedback loop generates a bistable system with irreversible activation of apoptosis at excessive levels of cellular stress [52,57]. This bistability would be important by separating the survival and cell death processes during oxidative stress. However, the verification of the presence and the importance of this regulatory loop upon oxidative stress requires further experimentally support.

Here we want to confirm that our goal was to give a general dynamical description of NRF2-dependent oxidative stress response mechanism. Although oxidative stress induces autophagy in many cells, these results are controversial in several cellular systems (e.g., astrocytes act differently). Corresponding to the controversial data we must admit that generalization of our statements is difficult. There are some special cases when the activation profile of autophagic process is completely different with respect to oxidative stress. e.g., both modest and intermediate levels of oxidative stress induced by various toxins, such as paraquat or rotenone, can inhibit autophagy without inducing cell death [58]. However, the exact role of NRF2 is not known yet. 

## 5. Conclusions

Understanding how NRF2 regulates cell survival, in particular autophagy, is highly relevant in several oxidative stress related diseases, such as neurodegenerative diseases, metabolic diseases, inflammation, carcinogenesis and chronic obstructive pulmonary disease [59]. Interestingly, both NRF2 and autophagy are considered as double-edged swords in cancer due to their ambiguous role in promoting cell survival in healthy and cancerous cells [60,61]. As oxidative stress is a key stressor in different phases of carcinogenesis regulating NRF2 and autophagy separately, and as we pointed out, in a coordinated manner, the proposed combined model highlights the difficulties of pharmacological targeting of this system. Nevertheless, such computational models can efficiently contribute to smart approaches against cancer and other complex diseases [62,63]. Only by modeling and experimentally validating intertwined feedback and feedforward loop systems, we can translate our improved understanding to promote advanced therapies against complex diseases.

## Supplementary Materials

The following are available online at http://www.mdpi.com/2076-3921/7/3/39/s1, Document S1: The importance of systems-level feedbacks of NRF2-controlled autophagy upon oxidative stress.
