# Supplementary material for: Systems-Level Feedbacks of NRF2 Controlling Autophagy upon Oxidative Stress Response
Source: Antioxidants (Basel). 2018 Mar 5;7(3):39. doi: 10.3390/antiox7030039 (PMC5874525; doi:10.3390/antiox7030039)
Supplement: Supplementary file 1 [file antioxidants-07-00039-s001.pdf]

The importance of systems-level feedbacks of NRF2-controlled autophagy upon oxidative stress

1. XPP codes for computer simulations

1.1. The code for visualizing NRF2-AMPK balance curves

```
# a model to generate the balance curves of NRF2 and AMPK with XPP-AUT

# initial conditions
# NRF2=0, AMPK =0

# differential equations
# NRF2 represents the active form of NRF2 protein
NRF2' = (kanrf + kanrf"*AMPK)*(NRF2T- NRF2)/(Jnrf + NRF2T- NRF2) - (kinrf +
kinrf'*mTOR)*Nrf2/(Jnrf + NRF2)

# AMPK represents the active form of AMPK protein
AMPK' = kaak*mRNA*(AMPKT-AMPK)/(Jampk + AMPKT-AMPK) - kiak*AMPK/(Jampk +
AMPK)

# steady state functions
# mRNA represents the mRNA level of AMPK
mRNA = (kamr + kamr'*stress/(1+stress))*mRNAT/(kamr +
kamr'*stress/(1+stress) + kimr + kimr'*NRF2)

# mTOR represents the active form of mTORC1 complex
mTOR = (kamtor + kamtor'*stress/(1+stress))* mTORT/(kamtor +
kamtor'*stress/(1+stress) + kimtor + kimtor'*AMPK + kimtor"*NRF2)

# AUTA represents the active form of autophagy inducer
AUTA = AUUT*GK(kaau + kaau'*NRF2 + kaau"*AMPK,kiau + kiau'*mTOR +
kiau"*DEATH,Jau,Jau)

# DEATH represents the active form of cell death inducer
DEATH = GK(kade + kade'*NRF2 + kade"*stress,kide + kide'*AUTA,Jdea,Jdea)

# 'Goldbeter-Koshand' function (GK)
GB(arg1,arg2,arg3,arg4) = arg2-arg1+arg2*arg3+arg1*arg4
GK(arg1,arg2,arg3,arg4) =
2*arg1*arg4/(GB(arg1,arg2,arg3,arg4)+sqrt(GB(arg1,arg2,arg3,arg4)^2-
4*(arg2-arg1)*arg1*arg4))

# parameters at physiological conditions
p stress=0
p kanrf=0.05, kanrf"=2, kinrf=0.01, kinrf'=15, jnrf=0.001, Nrf2T=1
p kamr=0.02, kamr'=1, kimr=0.25, kimr'=20, mRNAT=1
p kaak=7.5, kiak=1.5, AMPKT=1, Jampk=0.2
p kamtor=0.05, kamtor'=0.03, kimtor=0.01, kimtor'=0.5, kimtor"=10, mTORT=1
p AUUT=1, kaau=0.075, kaau'=0.001, kaau"=3, kiau=0.1, kiau'=10, kiau"=20,
Jau=0.2
p kade'=0.5, kade"=0.05, kide=0.01, kide'=30, Jdea=0.1

# simulating low level of oxidative stress: stress=0.1
# simulating intermediate level of oxidative stress: stress=0.5
# simulating high level of oxidative stress: stress=5
# simulating AMPK hyper-activation: kaak=45
# simulating AMPK inactivation: AMPKT=0.1
# simulating mTOR inactivation: mTORT=0.1
# simulating NRF2 down-regulation: NRF2T=0.1
```

```
done
```

1.2. The code for visualizing NRF2-autophagy inducer balance curves

```
# a model to generate the balance curves of NRF2 and autophagy inducer
(AUTA) with XPP-AUT

# initial conditions
# NRF2=0, AUTA =0

# differential equations
# NRF2 represents the active form of NRF2 protein
NRF2' = (kanrf + kanrf"*AMPK)*(NRF2T- NRF2)/(Jnrf + NRF2T- NRF2) - (kinrf +
kinrf'*mTOR)*Nrf2/(Jnrf + NRF2)

# AUTA represents the active form of autophagy inducer
AUTA' = (kaau + kaau'*NRF2 + kaau"*AMPK)*(AUUT-AUTA)/(Jau + AUUT-AUTA) -
(kiau + kiau'*mTOR + kiau"*DEATH)*AUTA /(Jau + AUTA)

# steady state functions
# mRNA represents the mRNA level of AMPK
mRNA = (kamr + kamr'*stress/(1+stress))*mRNAT/(kamr +
kamr'*stress/(1+stress) + kimr + kimr'*NRF2)

# AMPK represents the active form of AMPK protein
AMPK = AMPKT*GK(kaak*mRNA,kiak,Jampk,Jampk)

# mTOR represents the active form of mTORC1 complex
mTOR = (kamtor + kamtor'*stress/(1+stress))*mTORT/(kamtor +
kamtor'*stress/(1+stress) + kimtor + kimtor'*AMPK + kimtor"*NRF2)

# AUTA represents the active form of autophagy inducer
AUTA = AUUT*GK(kaau + kaau'*NRF2 + kaau"*AMPK,kiau + kiau'*mTOR +
kiau"*DEATH,Jau,Jau)

# DEATH represents the active form of cell death inducer
DEATH = GK(kade + kade'*NRF2 + kade"*stress,kide + kide'*AUTA,Jdea,Jdea)

# 'Goldbeter-Koshand' function (GK)
GB(arg1,arg2,arg3,arg4) = arg2-arg1+arg2*arg3+arg1*arg4
GK(arg1,arg2,arg3,arg4) =
2*arg1*arg4/(GB(arg1,arg2,arg3,arg4)+sqrt(GB(arg1,arg2,arg3,arg4)^2-
4*(arg2-arg1)*arg1*arg4))

# parameters at physiological conditions
p stress=0
p kanrf=0.05, kanrf"=2, kinrf=0.01, kinrf'=15, jnrf=0.001, Nrf2T=1
p kamr=0.02, kamr'=1, kimr=0.25, kimr'=20, mRNAT=1
p kaak=7.5, kiak=1.5, AMPKT=1, Jampk=0.2
p kamtor=0.05, kamtor'=0.03, kimtor=0.01, kimtor'=0.5, kimtor"=10, mTORT=1
p AUUT=1, kaau=0.075, kaau'=0.001, kaau"=3, kiau=0.1, kiau'=10, kiau"=20,
Jau=0.2
p kade'=0.5, kade"=0.05, kide=0.01, kide'=30, Jdea=0.1

# simulating low level of oxidative stress: stress=0.1
# simulating intermediate level of oxidative stress: stress=0.5

done
```

1.3. The code for computer simulations of oxidative stress response mechanism

```
# a model to generate computer simulation of cellular oxidative stress
response mechanism with XPP-AUT

# initial conditions
init  NRF2=3.187*10-5,  mRNA=0.074,  AMPK=0.087,  mTOR=0.482,  AUTA=0.012,
DEATH=6.991*10-6

# differential equations
# NRF2 represents the active form of NRF2 protein
NRF2' = (kanrf + kanrf"*AMPK)*(NRF2T- NRF2)/(Jnrf + NRF2T-NRF2) - (kinrf +
kinrf'*mTOR)* NRF2/(Jnrf + NRF2)

# mRNA represents the mRNA level of AMPK
mRNA'  =  (kamr  +  kamr'*stress/(1+stress))*(mRNAT-mRNA)  -  (kimr  +
kimr'*NRF2)*mRNA

# AMPK represents the active form of AMPK protein
AMPK' = kaak*mRNA*(AMPKT-AMPK)/(Jampk + AMPKT-AMPK) - kiak*AMPK/(Jampk +
AMPK)

# mTOR represents the active form of mTORC1 complex
mTOR'  =  (kamtor  +  kamtor'*stress/(1+stress))*(mTORT-mTOR)  -  (kimtor  +
kimtor'*AMPK + kimtor"*NRF2)*mTOR

# AUTA represents the active form of autophagy inducer
AUTA' = (kaau + kaau'*NRF2 + kaau"*AMPK)*(AUUT-AUTA)/(Jau + AUUT-AUTA) -
(kiau + kiau'*mTOR + kiau"*DEATH)*AUTA /(Jau + AUTA)

# DEATH represents the active form of cell death inducer
DEATH' =(kade'*NRF2 + kade"*stress)*(1-DEATH)/(Jdea + 1-DEATH) - (kide +
kide'*AUTA)*DEATH/(Jdea + DEATH)

# parameters at physiological conditions
p stress=0
p kanrf=0.05, kanrf"=2, kinrf=0.01, kinrf'=15, jnrf=0.001, Nrf2T=1
p kamr=0.02, kamr'=1, kimr=0.25, kimr'=20, mRNAT=1
p kaak=7.5, kiak=1.5, AMPKT=1, Jampk=0.2
p kamtor=0.05, kamtor'=0.03, kimtor=0.01, kimtor'=0.5, kimtor"=10, mTORT=1
p AUUT=1, kaau=0.075, kaau'=0.001, kaau"=3, kiau=0.1, kiau'=10, kiau"=20,
Jau=0.2
p kade'=0.5, kade"=0.05, kide=0.01, kide'=30, Jdea=0.1

# simulating low level of oxidative stress: stress=0.1
# simulating intermediate level of oxidative stress: stress=0.5
# simulating high level of oxidative stress: stress=5
# simulating AMPK hyper-activation: kaak=45
# simulating AMPK inactivation: AMPKT=0.1
# simulating mTOR inactivation: mTORT=0.1
# simulating NRF2 down-regulation: NRF2T=0.1
# simulating the diminish of NRF2 ┤ mTOR: kimtor"=0
# simulating the diminish of mTOR ┤ NRF2: kinrf'=0

done
```

1.4. The code for visualizing signal response curve

```
# a model to generate a signal response curve of cellular oxidative stress
response mechanism with XPP-AUT

# initial conditions
init  NRF2=3.187*10-5,  mRNA=0.074,  AMPK=0.087,  mTOR=0.482,  AUTA=0.012,
DEATH=6.991*10-6

# differential equations
# NRF2 represents the active form of NRF2 protein
NRF2' = (kanrf + kanrf"*AMPK)*(NRF2T- NRF2)/(Jnrf + NRF2T-NRF2) - (kinrf +
kinrf'*mTOR)* NRF2/(Jnrf + NRF2)

# mRNA represents the mRNA level of AMPK
mRNA'  =  (kamr  +  kamr'*stress/(1+stress))*(mRNAT-mRNA)  -  (kimr  +
kimr'*NRF2)*mRNA

# AMPK represents the active form of AMPK protein
AMPK' = kaak*mRNA*(AMPKT-AMPK)/(Jampk + AMPKT-AMPK) - kiak*AMPK/(Jampk +
AMPK)

# mTOR represents the active form of mTORC1 complex
mTOR'  =  (kamtor  +  kamtor'*stress/(1+stress))*(mTORT-mTOR)  -  (kimtor  +
kimtor'*AMPK + kimtor"*NRF2)*mTOR

# AUTA represents the active form of autophagy inducer
AUTA' = (kaau + kaau'*NRF2 + kaau"*AMPK)*(AUUT-AUTA)/(Jau + AUUT-AUTA) -
(kiau + kiau'*mTOR + kiau"*DEATH)*AUTA /(Jau + AUTA)

# DEATH represents the active form of cell death inducer
DEATH' =(kade'*NRF2 + kade"*stress)*(1-DEATH)/(Jdea + 1-DEATH) - (kide +
kide'*AUTA)*DEATH/(Jdea + DEATH)

# parameters at physiological conditions
p stress=0
p kanrf=0.05, kanrf"=2, kinrf=0.01, kinrf'=15, jnrf=0.001, Nrf2T=1
p kamr=0.02, kamr'=1, kimr=0.25, kimr'=20, mRNAT=1
p kaak=7.5, kiak=1.5, AMPKT=1, Jampk=0.2
p kamtor=0.05, kamtor'=0.03, kimtor=0.01, kimtor'=0.5, kimtor"=10, mTORT=1
p  AUUT=1, kaau=0.075, kaau'=0.001, kaau"=3, kiau=0.1, kiau'=10, kiau"=20,
Jau=0.2
p kade'=0.5, kade"=0.05, kide=0.01, kide'=30, Jdea=0.1

done
```

2. Introducing the bioinformatical analysis of phosphorylation site search on NRF2

PhosphoSite Plus (https://www.phosphosite.org/proteinAction?id=564&showAllSites=true) shows the preferred phosphorylation site of mTOR kinase:

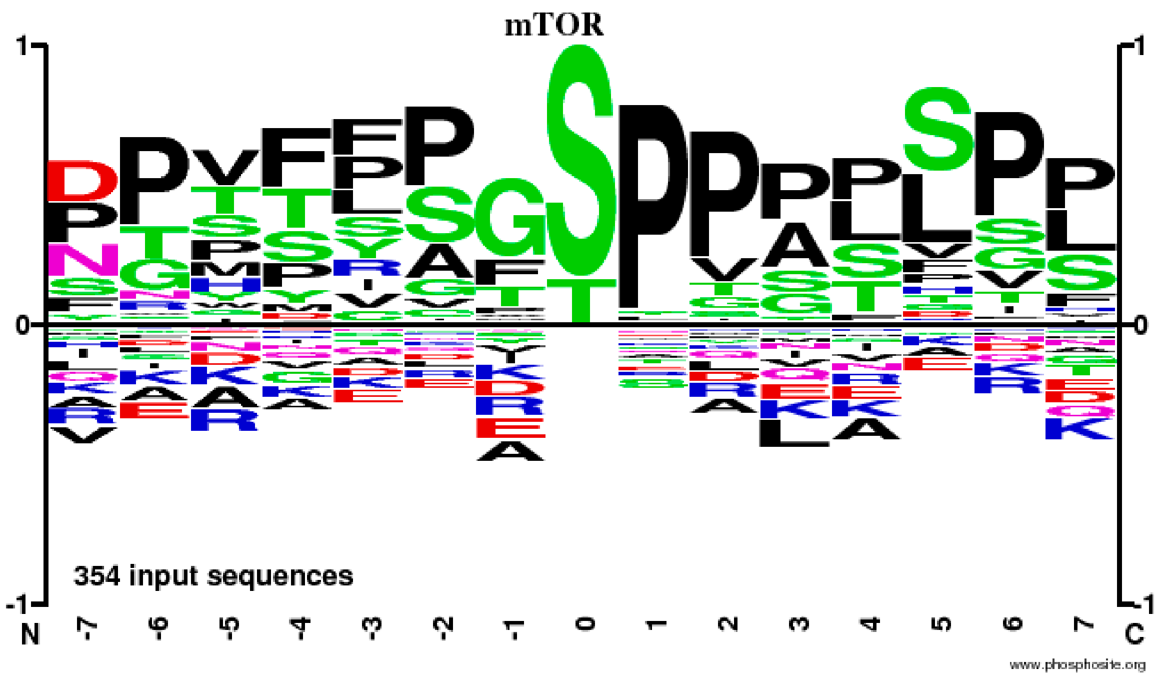

Using NetPhos 3.1. (http://www.cbs.dtu.dk/services/NetPhos/) the potential serine phosphorylation sites were searched in human NRF2 protein sequence (the algorithm of this freely availably software can be found in Blom et al., 2014). The program identified two phosphorylation sites with the following amino acid sequences:

- Ser-351: SLNTSPSVA
- Ser-356: PSVASPEHS

where the yellow colour shows that this amino acid around the potential Ser resiude is preferred by mTOR kinase. According to analysis done by NetPhos 3.1., the protein kinase of these phosphorylation sites are yet unidentified.
